# 

**Published:** 1878-11

**Authors:** 


					﻿NOVEMBEB, 1878.
TWENTY-FIFTH YEAR—N<> 299.
HALL’S
Journal of Health.
PUBLISHED AT 74 FOURTH AVENUE,
inte-w Srcxrac-
E. H. GIBBS, A.M., M.D., Editor.
Medical Office—139 East Eighth Street, Near Broadway.
OFFICE HOURS—FROM 10 A.M. TO 3 P.M.
AGENTS FOR THE TRADE:
AMERICAN NEWS COMPANY AND NEW YORK NEWS COMPANY.
Terms, $1.50 a Year, or $1.00 for Eight Months. Postage paid.
SINGLE NUMBER, 15 CENTS.
Couch & Johnston, Printers, 11 Frankfort Street, New York.
				

